# Identification of High‐Yielding and Drought‐Tolerant Melon Genotypes Based on Yield–Trait Combinations: A Comparison of Pcor Index and GYT Biplot Approaches

**DOI:** 10.1002/fsn3.70048

**Published:** 2025-02-28

**Authors:** Mohammad Reza Naroui Rad, Behnam Bakhshi, Ahmad Ghasemi, Mahmoud Mohammad Ghasemi, Jahangir Abbasi Kohpalekani

**Affiliations:** ^1^ Horticulture Crops Research Department Sistan Agricultural and Natural Resources Research and Education Center, AREEO Zabol Iran; ^2^ Seed and Plant Improvement Institute Agricultural Research, Education and Extension Organization (AREEO) Karaj Iran

**Keywords:** drought tolerance, GYT biplot, high yielding, Pcor index

## Abstract

Melon is a spring plant that grows well in Iran's warm, arid climate, so it is in high demand during the summer season. However, the genotype‐by‐environment interaction (GEI) effect and the difficulty of choosing several agronomic characteristics make breeding high–yielding melon cultivars difficult. This study utilized a novel methodology for genotyping that incorporates several characteristics, employing the genotype by yield by trait (GYT) biplot method. This technique uses yield–trait combinations to rate genotypes and highlight their strengths and weaknesses. Furthermore, the findings demonstrated that genotypes with various combinations of yield and trait can be found and employed to enhance genetic material in melon breeding programs. The best genotypes were 23, 13, and 8, as they exhibited an ideal combination of plant output under drought stress circumstances and agro‐physiological traits. The most superior genotype, genotype number 23, can be suggested for breeding operations based on the Pcor index and the GYT biplot analysis. This study shows the usefulness and effectiveness of the recently introduced Pcor and GYT biplot, which can assist breeders in choosing the best cultivars for high‐yielding and drought‐tolerant melon genotypes.

## Graphical abstract

The breeding lines 23, 13, and 8 were the best ones, as they had the best combination of agro‐physiological characteristics and plant yield under drought stress conditions. Based on the Pcor index and the GYT biplot analysis, genotype number 23 was the best one.

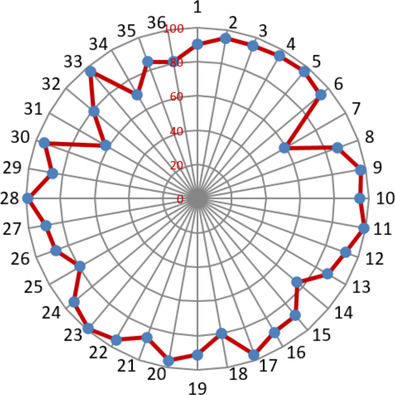

## 1 Introduction

Breeders' experience and genetic diversity within a species are key factors in the selection process that occurs in breeding programs. Plant improvement depends significantly on morphological, molecular, and biochemical techniques (Kandel et al. 2018). The principal origin of this melon group is *Cucumis melo* L. (2n = 2× = 24), which has historically been cultivated across various Iranian regions (Munger and Robinson 1991). Regarding the quantity of melon produced worldwide, Iran is ranked third (FAO 2018). One notable environmental limitation that has a negative impact on plant survival, growth, and production is drought stress. Evaluating yield stability is crucial for determining a plant's ability to tolerate drought stress (Guttieri et al. 2001) because climate change and global warming are major agricultural concerns (Shi et al. 2021).

Due to the complex factors that influence plant behavior under field conditions, such as plant growth stage, stress intensity, time and duration of stress, and the physical and chemical characteristics of soil and their interactions, breeding high‐yielding melon cultivars is difficult. Abiotic stress screening of melon genotypes requires certain morphological traits, including fruit yield, flesh and skin weight, cavity diameter, and fruit weight (Naroui Rad and Bakhshi 2021). Fruit weight may also be affected by the genotype‐by‐environment interaction (GE), which can be utilized to increase yield but may also make selection more difficult because it is dependent on a variety of environmental parameters, including the amount and distribution of rainfall, the seasons, and the years (Mousavi et al. 2023). Breeders can employ a variety of models and techniques to overcome these obstacles and assess genotypes' stability and yield under various conditions (Vaezi et al. 2019).

Independent culling is one of the models by which genotypes that do not achieve a trait's minimum needed value are eliminated, independent of how well the genotypes perform in other characteristics. Another model is index selection, in which genotypes are ranked based on the index values of a linear combination of the target traits (Sofi et al. 2022; Yan and Frégeau‐Reid 2018). Nonetheless, there are certain limitations to both models, including their high subjectivity and the possibility of weight and truncation point variations over time and among researchers, even for a similar dataset (Yan and Frégeau‐Reid 2018). Consequently, in order to choose the optimal melon genotypes based on a variety of traits, alternative techniques are required.

Interaction between the genotype and trait (GT) as well as triple interaction genotype by yield by trait (GYT) will analyze as graphical, which shows the relationships between the genotype and trait in an understandable and visual manner. In order to classify and assess genotypes and determine the critical characteristics influencing plant production in other crops, including barley, soybean, corn, wheat, sorghum, and rapeseed, the GT biplot method has been implemented by researchers (Shojaei et al. 2020; Mukondwa et al. 2021; Zulfiqar et al. 2021; Santana et al. 2021; Santos et al. 2021; Bakhshi et al. 2023a, 2023b; Bakhshi and Shahmoradi 2023; Amiri Oghan et al, 2024). However, the cumulative impact of all other characteristics on yield cannot be determined using this method. Recently, the GYT biplot methodology has been developed to address this limitation by determining the combined effect of other characteristics on yield. The superiority index (SI), which is a useful measure that visually represents the advantages and disadvantages of a given genotype for every characteristic that affects yield, is provided by the methodology (Yan and Frégeau‐Reid 2018; Kendal 2019). The utilization of this approach in melon selection programs is novel and is regarded as an innovative component of the present investigation. One of the most commonly used statistical techniques for the ordination and dimensionality reduction of multivariate datasets in a variety of scientific disciplines is principal component analysis (PCA). As a correlation between characteristic scores in cultivars and the first component in PCA, the Pcor index is released for the first time in this paper, and this index was followed by PCA. Many researchers are currently working on various selection strategies for genotypes that are resistant to drought by using susceptibility and/or tolerance indices. The Pcor is being introduced here and can assist breeders in choosing the best cultivars. This study aims to identify genotypes with the most desirable characteristics for different characteristics, investigate relationships between the analyzed traits, and classify genotypes based on the studied traits. This is accomplished by examining the impact of genotype traits using the GT biplot approach. Additionally, the study compares and assesses the effectiveness of the GT biplot, GYT biplot, and Pcor index approaches in terms of choosing superior melon genotypes based on a variety of features.

## 2 Material and Methods

### 2.1 Plant Material and Experimental Design

The study was carried out in Southeast Iran at the Zahak Agricultural Research Station (31°54′ N; 61°41′ E, 483 m above sea level). The station experiences nearly 53 mm of yearly rainfall and 4000–4500 mm∙year^−1^ of evaporation. The soil type was sandy loam. Thirty‐six muskmelon genotypes (Table 1) were assessed in the presence of water depletion after being obtained from the National Plant Gene Bank of Iran. Nutrition was applied prior to sowing, consisting of 80 kg ha^−1^ of P_2_O_5_ and 100 kg∙ha^−1^ of N from urea. Every hole was seeded with two seeds, spaced 0.5 × 0.5 m apart. Each genotype consisted of six plants and two replications in a square lattice pattern. Replications were spaced 2 m apart, and rows were separated by 0.5 m. Every plant was regarded as a replication.

**TABLE 1:** Origins, accession numbers, and the numbers of studied genotypes.

Origin	Genotype	No.	Genotype	No.	Origin	Genotype	No.
Iran	KC‐357009	25	TN‐628	13	Iran	TN‐345	1
Iran	KC‐357067	26	TN‐92319	14	Iran	KC‐35700	2
Iran	KC‐357100	27	TN623	15	Iran	TN‐271	3
Iran	TN‐278	28	TN92317	16	Iran	KC‐357250	4
Iran	TN‐272	29	TN92312	17	Iran	KC‐357238	5
Iran	KC‐357104	30	TN622	18	Iran	TN‐441	6
Iran	KC‐357079	31	KC357020	19	Iran	TN‐92401	7
Iran	KC‐357063	32	TN621	20	Iran	TN377	8
Iran	KC‐357047	33	TN‐92306	21	Iran	TN‐277	9
Iran	KC‐357062	34	TN‐92302	22	Iran	KC‐257236	10
Iran	Check‐Sefidak	35	KC357044	23	Iran	TN629	11
Iran	Check‐Suski	36	KC357154	24	Iran	TN92334	12

### 2.2 Irrigation and Pest Management

On the basis of the time‐domain reflectometry (TDR) data, irrigation was applied when 25%, 50%, or 75% of the available water in the soil had been depleted. At a depth of 50 cm in the soil, two TDR probes (CS616; Campbell Sci., Ettlingen, Germany) were positioned at every irrigation level. In both years, furrow irrigation was started in March and continued every 10 days. Two applications of diazinon were made, separated by 10 days, to prevent melon flies, beginning with the formation of fruit. Weeds were manually controlled. After ripening, the fruits of each genotype's plant were harvested and weighed.

### 2.3 Studied Traits

At fruit ripening, 14 traits were assessed, including physiological traits relevant to water‐deficit conditions and yield components. These traits included: fruit number per plant (NF); weight of fruits (FW); length of fruits (FL); width of fruits (F.WI); total soluble solids (TSS); single plant yield (SPY); diameter of flesh (FLD); diameter of cavity (CD); length of plant (PL); chlorophyll content index (CHL), determined by SPAD502 portable chlorophyll meter (Minolta Ltd.); canopy temperature (CT), measured using an infrared thermometer positioned about 50 cm above the canopy and 0.5–1 m from the edge of the plot; relative water content (RWC = [{W–DW}/{TW–DW}] × 100), where W represents sample fresh weight, TW represents sample turgid weight, and DW represents sample dry weight, Days to maturity (DM); and Length of root (RL), determined on the primary root following the removal of soil.

### 2.4 Statistical Analysis

SAS was used to conduct the statistical analysis, and the homogeneity of residual variance was confirmed by the Bartlett's test. Data normality was examined by utilizing the Shapiro–Wilk test in SPSS software (Ver. 19). A combined ANOVA was performed using the GLM (general linear model) function in SAS, treating year and genotype as random and fixed effects, respectively.

The GGEbiplot program was used to carry out the biplot analyses for GT and GYT (Yan and Rajcan 2002; Yan and Frégeau‐Reid 2018). The first two major components of the original data, as determined using singular value decomposition (SVD), generated graphical biplots for the interaction between GT. The first and second principal components of the data converted by multiplying or dividing by the grain yield (GY) and acquired from the SVD were used to create the biplots for GYT. Whether a large or small numerical number was preferred for each characteristic determined the transformation. The numerical values were multiplied by GY for features that positively impacted yield, such as seed weight, number of seeds per head, and stem and head diameter. The values were split by GY for characteristics that negatively impacted yield, such as height of plant and number of days to flowering and ripening. The interrelationships between agricultural characteristics were examined using the biplot analysis results, and appropriate melon genotypes were found based on a set of desirable traits. For every combination of yield by characteristic, the SI was calculated using the standardized GYT (Yan and Frégeau‐Reid 2018). The correlation between cultivar scores in PCA and the first component resulted in the Pcor index. The optimal genotypes were chosen using the trend line. The correlation coefficients of cultivar scores with characteristics in all cultivars can be an excellent indicator for determining tolerant and high‐yielding cultivars because PCA is one of the most appropriate analyses to determine characteristics that justify the greatest amount of variance among cultivars.

## 3 Results

### 3.1 Analysis of Variance

The comprehensive analysis of variance demonstrated significant impacts of genotypes, the environment, and their interaction (Table 2). Significant genotype (G) effects were seen for all the examined characteristics. The mean values of all studied characteristics are presented in Table 3.

**TABLE 2:** Analysis of variance for plant yield.

Source of variation	df	SS	MS	Explained (%)
Treatments	107	1.24E + 08	1159117**	
Genotypes	35	17654806	504423**	14
Environments	2	97575700	48787850**	78
Block	3	129308	43103	
Interactions	70	8795054	125644**	7
IPCA1	32	86662052	2708189**	69
IPCA2	30	21133001	704433**	17
Residue	8	1000002	125000	
Total	215	1.27E + 08		

**significant at 0.01 statistical level.

**TABLE 3:** Average of measured traits in 36 melon genotypes.

No	N.F	F.W	F.L	F.WI	SUGAR	S.P.Y	FL.D	C.D	P.L	SPAD	C.T	RWC	D.M	R.L
1	2	2822.5	24	17.25	5	4395	8.3	3.6	193	72	33.5	0.6	87.5	38.5
2	2	2105	26	14.25	8	3335	8.6	4.15	203	69.5	35	0.7	85.5	42.5
3	2	2495	31	14	4	4010	7.6	3.7	218	64	32	0.7	85.5	47
4	2.5	1625	20	15.5	5.3	3320	8.1	2.95	193	62	33.5	0.6	87	35
5	1.5	1550	26	12.75	6	1820	7.6	3.55	163	62.5	36	0.6	86	35
6	2	2525	21	16	7	3630	9.65	5.05	155.5	85.5	32	0.7	83.5	37.5
7	1.5	1475	19	13	5	1745	8.45	3.05	183	68.5	32.5	0.6	86	32
8	1.5	2560	28	15	5.3	2830	8.15	4.35	153	72	38.5	0.6	86.5	40.5
9	2.5	2485	26.5	16	4.8	5040	9.2	4.05	243	67	33.5	0.7	84	41.5
10	1.5	1530	18.5	12.5	5	1800	8.45	3.7	188	70	32	0.6	90.5	41.5
11	2	1810	26	13	9.3	2780	7.55	3.65	213	69.5	33.5	0.6	91	44.5
12	2	1790	28	13	6.8	2805	7.85	3.95	123	80.5	35.5	0.6	83.5	32.5
13	2	2198.5	27	14.5	8	3958.5	6.95	3.2	213	69.5	34.5	0.6	86	42.5
14	2	1806	20.5	15	8.8	2802	8.55	3.7	238	78	33	0.7	83.5	47
15	2	2215	19.5	16	8.5	3660	9.15	3.9	203	75	31	0.7	82	43.5
16	2	2065	26	14.5	7	3500	8.2	3.8	173	62	35.5	0.6	86.5	33
17	2	1842.5	25	17	4.5	2815	8.45	4.4	188	58.5	36	0.5	87	31
18	2.5	1860	24.5	15	7.3	3790	8.15	3.4	153	75.5	31.5	0.7	85	40
19	2	840	15	12.5	5	1470	8.15	3.9	198	65.5	32.5	0.8	87	29.5
20	2.5	1010	19.5	11	9.8	2090	7.3	2.9	188	79.5	33.5	0.7	86.5	32
21	2	1221.5	21	14	4	1963	8.45	3.3	203	52.5	37	0.6	88.5	36
22	2	1820	19.5	8	6.8	3015	9.95	3.4	218	67	32.5	0.7	89.5	40
23	2	3927.5	35	17	4.8	6242.5	8.5	3.7	223	64.5	32	0.7	87	40
24	1.5	1030	19	12.5	9	1300	8.05	3.1	188	71	32	0.6	84	30
25	2.5	1420	20.5	16	8	2910	9.1	4	203	65	33	0.6	86	29.5
26	3	890	15.5	12.5	5.5	2115	8.15	2.8	113	64	34	0.6	84	31
27	2	1305	20	14.75	3.3	2130	8.4	3.35	183	59.5	32	0.7	85	39
28	1.5	2195	16	14.5	8.8	2465	8.1	3.4	180.5	66	33.5	0.5	84.5	30.5
29	2.5	1350	24	15	4.8	2770	8.05	3.5	233	65	33.5	0.7	87	33.5
30	2.5	2365	29	14.5	7.8	4800	8.7	3.75	218	74	30.5	0.8	87.5	30.5
31	2.5	780	16	11.5	8.5	1630	7.7	2.85	148	57.5	30	0.7	82	42.5
32	3	695	13	10.5	9.3	1655	6.2	2.9	183	71	37	0.6	83	40.5
33	1.5	1360	17	13.5	3.3	1630	8.4	4.25	143	64	35.5	0.6	86.5	38.5
34	2.5	1169.5	19.5	11	3.8	2689.5	8.1	3.75	233	76.5	31.5	0.7	82	50.5
35	2.5	852.5	16	12.25	8.5	1775	8.6	3.2	238	56.5	31.5	0.7	82	57
36	2	998	18.5	13	6	1646	7.65	3.75	200.5	64.5	35.5	0.7	82.5	40.5

### 3.2 The GT Biplot for Profiling Melon Traits

**FIGURE 1:**
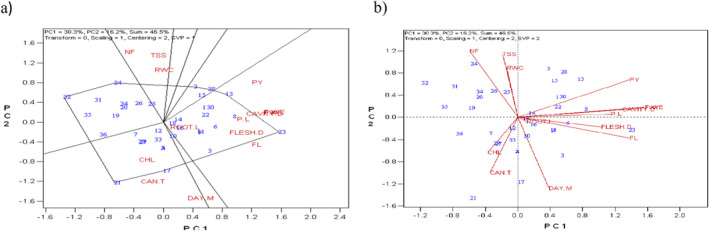
(a) Polygon view of the genotype × trait biplot of melon genotypes. (b) Vector view of the genotype × trait biplot of melon genotypes.

The findings of the GT biplot analysis revealed that, with a total of 46.5%, the first two principal components accounted for 30.3% and 16.2% of the variability of the standardized data (Figure 1). Using the polygon view is one of the most effective ways to use the GT biplot to find genotypes that excel in one or more characteristics. For the test characteristics, the superior genotypes are represented by the vertex genotypes in every sector of the polygon view. The vertex genotypes were 21, 32, 24, 28, and 23, as seen in Figure 1a. In terms of fruit length, flesh diameter, and plant yield, genotype 23 was the most preferred genotype.

**FIGURE 2:**
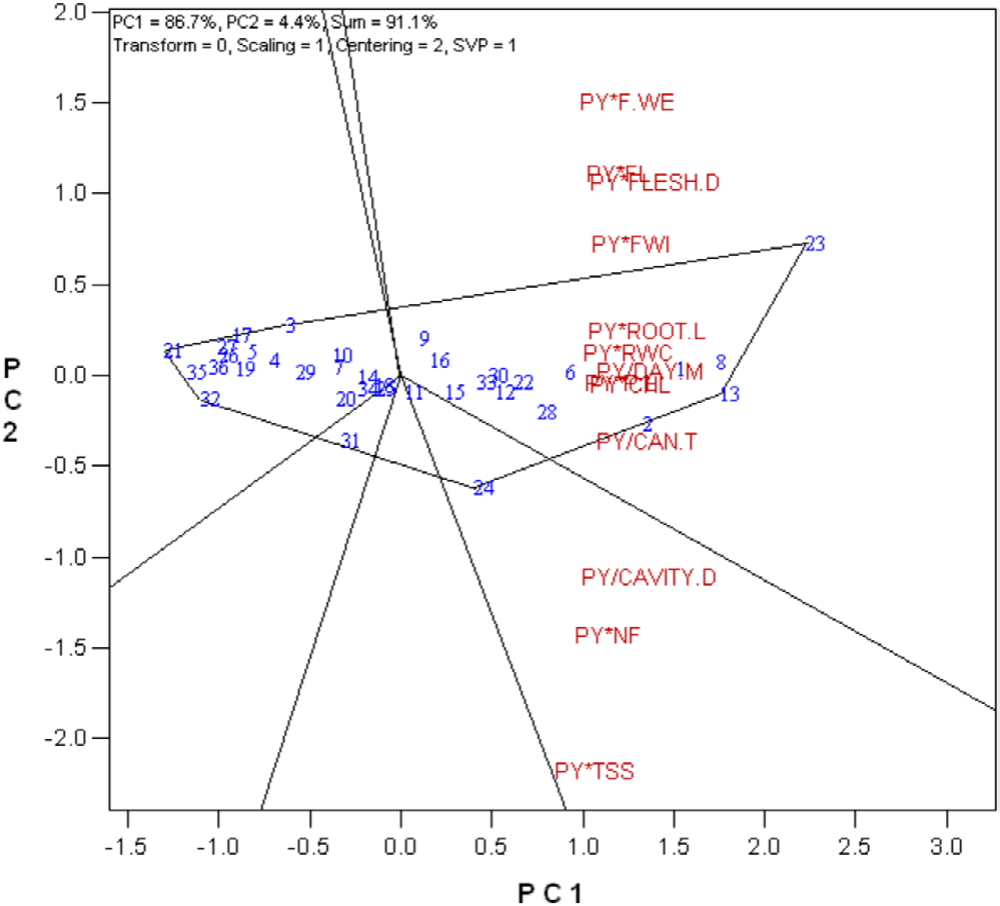
Polygon view of the genotype × yield × trait biplot of melon genotypes.

The GT biplot can also be used to analyze the relationships between characteristics using the vector view. The lines that link every characteristic to the origin in the vector view are referred to as vectors. The correlation between characteristics is shown by the angle between the vectors. Positive or negative correlations are implied by angles that are less than or greater than 90°, respectively. There is no correlation between the characteristics when the angles are near 90°. Strong positive connections were found, as illustrated in Figure 2, between chlorophyll and canopy temperature, diameter of flesh and length of fruit, diameter of cavity and weight of fruit, and yield of plant and weight of fruit. In terms of chlorophyll and canopy temperature, genotype 21 had the most significant values. For every characteristic that was measured, the vertex genotypes 32 and 24 performed worse.

### 3.3 The GYT Biplot for Melons' Classification

The genotypes with the greatest values for the combination of plant yield and other characteristics are displayed in the polygon view of the GYT biplot. The greatest values for PY*F.WE, PY*FLESH D, PY*ROOT.L, PY/CAN.T, PY*CHLL, PY/DAY.M, PY*RWC, and PY*FWI were found in genotypes 23 and 13. This suggests that these genotypes are superior when taking into account the total plant output in conjunction with the following factors: days to maturity, relative water content, width of fruit, weight of fruit, diameter of flesh, length of root, canopy temperature, and chlorophyll.

### 3.4 The GYT Biplot for Representation of the Yield–Trait Relationships

**FIGURE 3:**
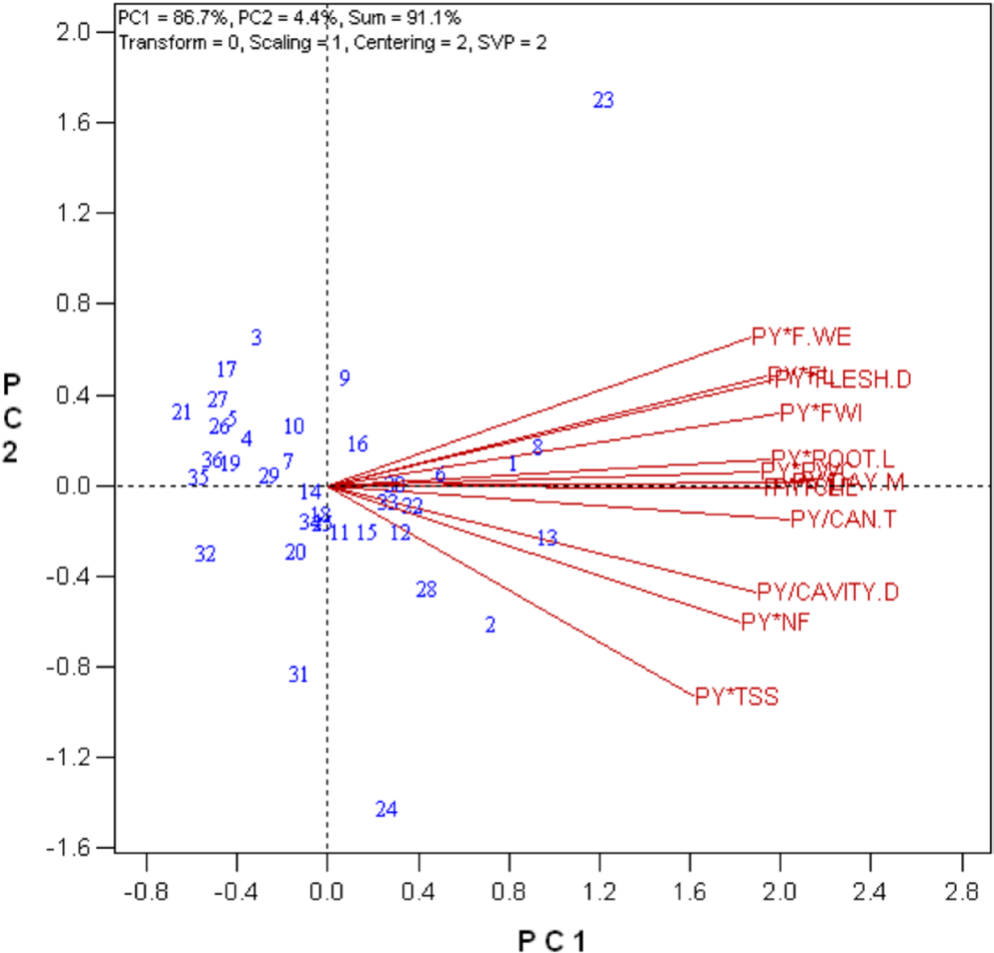
Vector view of the genotype × yield × trait biplot of melon genotypes.

The Figure 3 association between the combination of plant yield and other characteristics is displayed in the vector view of the GYT biplot. Strong positive correlations are indicated by the acute angles formed between the vectors. Significant correlations were observed between PY*ROOT.L and PY*RWC, as well as PY*FL, PY*FLESH.D, PY/CAVITY.D, and PY*NF.

### 3.5 Superiority vs. “Weaknesses and Strengths” of Genotypes

**FIGURE 4:**
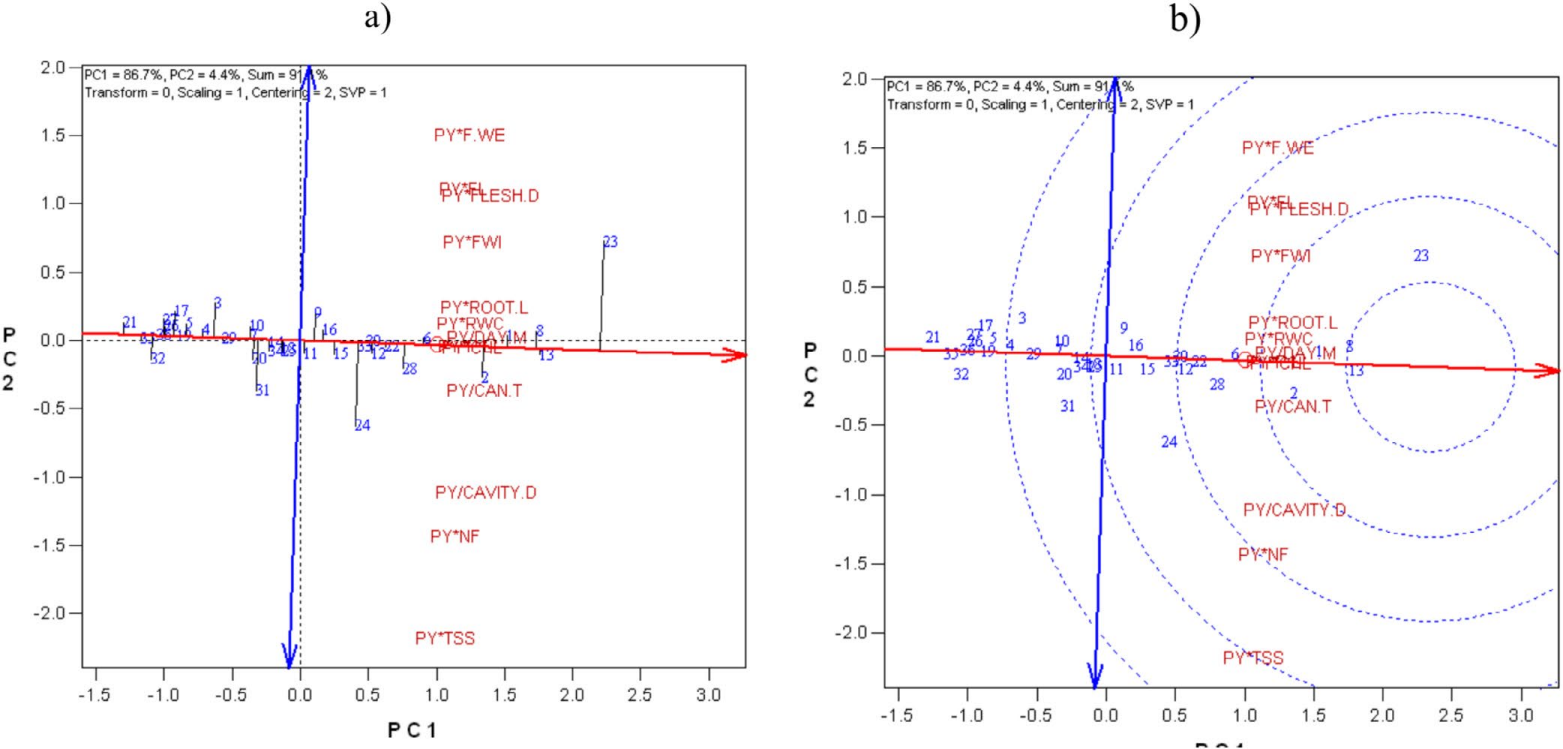
(a) Average tester coordinate (ATC) view of the genotype × yield × trait biplot to rank the genotypes based on overall superiority and their strengths and weaknesses. (b) Ranking traits based on both discriminating and representativeness.

GYT biplot expression using the average tester coordinate (ATC) showed that genotypes 23, 13, 8, 1, 2, and 6 were superior for all yield–trait combinations, whereas genotypes 21, 32, 27, 17, 5, 4, 3, 29, 10, 20, 31, 14, 34, and 25 were undesirable (Figure 4).

The original GT data (Table 2) and the grain yield (GY) data were used to conduct the GYT biplot analysis. Quantitative characteristics that affected yield positively were multiplied by GY, whereas characteristics that affected yield negatively (e.g., earliness characteristics) were divided by GY. In this sense, a superior yield–trait combination was indicated by a greater value in the GYT table. The data from the GYT table were shown graphically in the GYT biplot. With 86.7% and 4.4%, the first and second main components contributed 91% of the total variation in the GYT data. On the basis of their combinations of traits and yield, the genotypes were ranked using the mean × stability GYT biplot. The genotypes with above‐average yield–trait combinations were found on the right side of the double‐arrowed line. The genotypes that performed the best were 23, 13, 8, 1, 2, and 6. The most consistent and desired yield–trait combination was exhibited by genotype 13, which was positioned in the middle of the circles.

### 3.6 The Pcor Index for Selecting the Superior Genotype

**FIGURE 5:**
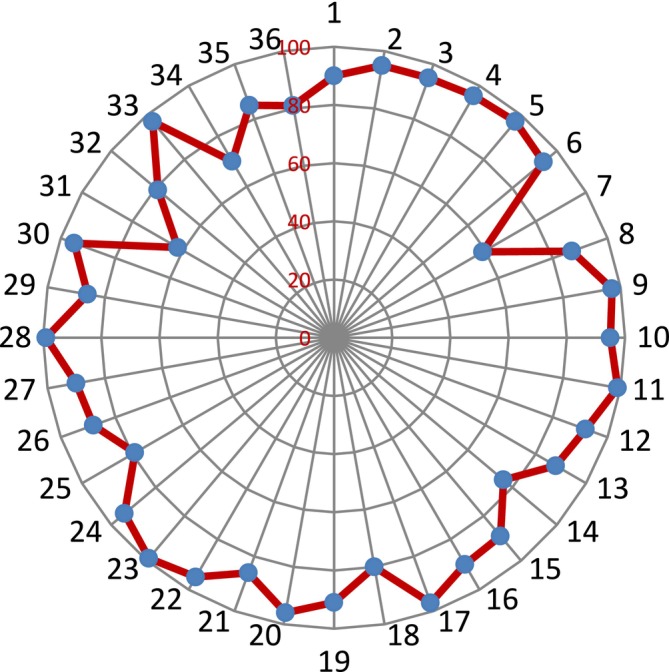
Pcor index as moving average trend for selection of the best genotypes.

On the basis of the Pcor index (Figure 5) and the GYT biplot analysis, genotype number 23 was the best one. Therefore, this genotype can be recommended for the breeding program according to both methods.

## 4 Discussion

Melon cultivation and breeding are essential for Iran, the demand for this fruit is high in summer. Moreover, melon is a spring plant that adapts well to the warm and arid regions of Iran (Naroui Rad et al. 2017). It is necessary to develop cultivars with high yield that are compatible with these regions. However, there are two main challenges in introducing new varieties. The first and most important one is the performance of the genotype in the special environment (GEI) effect, which affects the adaptability of the new genotypes in the target areas (Bakhshi et al. 2023a, 2023b). The next one is the breeding of the cultivars by a complexity of superiority in other agricultural traits besides yield.

Previous studies have shown that, based on the low heritability of yield, morphological, agronomical, and physiological traits must be considered in the selection process of the breeders for high‐yielding genotypes. Different methods have been proposed for the selection of suitable traits and yield at the same time, for example, GT and GYT, as biplots can depict graphical images and overviews of the original data. Compared with conventional methods, such as correlation coefficients or multivariate analysis, GT and GYT biplot methods are efficient statistical tools for visual evaluation, classification, and selection of suitable genotypes. Evaluation and grouping of genotypes with important traits by the GT biplot method have been used for other crops (Santos et al. 2021; Yan and Rajcan 2002; Malik et al. 2014; Santana et al. 2021). However, this method cannot identify the combined effect of all other traits on yield (Malik et al. 2014; Santana et al. 2021; Santos et al. 2021; Yan and Rajcan 2002). Due to this limitation, the method GYT was recently introduced; it graphically shows the combined effects of other traits on yield. This method provides an SI, which is an effective measure that graphically reveals the positive and negative aspects of a given genotype in terms of all yield‐affecting traits (Kendal 2019; Yan and Frégeau‐Reid 2018). This method has not been used previously in melon selection programs and is considered an innovative aspect of this study. In melon selection and breeding, this method has not been implemented before, so it is considered an innovative model. The GT method has been used previously, but the results of this research indicate that the GYT approach in melon selection is more efficient and useful. On the basis of the results and considering the combined effect of the investigated traits—fruit weight, cavity diameter, flesh diameter, and fruit length—on plant yield, genotypes 23, 13, 8, 1, and 2 were selected as the best genotypes in this study, whereas genotypes 21, 3, 32, 35, and 17 were identified as inferior genotypes. Using a set of useful traits for selection increases value in a cultivar, so genotypes 23, 13, and 8 are the top melons suggested for cultivation in locations like Sistan. The positive correlation of yield traits maximizes the efficiency of the GYT approach compared to the GT biplot and is an important feature of this method. This feature can reduce the cost of experiments in the melon breeding program and also lead to the reduction of traits in the selection process of genotypes by efficient traits. Regarding the results, the high correlation of cavity diameter with fruit weight makes one of these traits usable as the selection criterion.

## 5 Conclusion

This study applied the GYT biplot, a new approach to genotyping based on multiple traits. This approach is comprehensive and effective, as it ranks genotypes based on their yield–trait combinations and shows their strengths and weaknesses. The results also showed that contrasting genotypes can be identified based on the attributes for improving genetic material in melon breeding programs. The breeding lines 23, 13, and 8 were the best ones, as they had the best combination of agro‐physiological characteristics and plant yield under drought stress conditions. On the basis of the Pcor index and the GYT biplot analysis, genotype number 23 was the best one, and it can be recommended for the breeding programs according to both methods.

## Author Contributions

**Mohammad Reza Naroui Rad:** data curation (equal), methodology (equal), resources (equal), supervision (equal), writing – original draft (equal). **Behnam Bakhshi:** software (equal), writing – review and editing (equal). **Ahmad Ghasemi:** visualization (equal). **Mahmoud Mohammad Ghasemi:** visualization (equal). **Jahangir Abbasi Kohpalekani:** visualization (equal).

## Consent

Written informed consent was obtained from all study participants.

## Conflicts of Interest

The authors declare no conflicts of interest.

## Data Availability

The data that support the findings of this study are available from the corresponding author upon reasonable request.
